# A simple and rapid flow cytometry-based assay to identify a competent embryo prior to embryo transfer

**DOI:** 10.1038/srep39927

**Published:** 2017-01-06

**Authors:** Eva Pallinger, Zoltan Bognar, Jozsef Bodis, Timea Csabai, Nelli Farkas, Krisztina Godony, Akos Varnagy, Edit Buzas, Julia Szekeres-Bartho

**Affiliations:** 1 Department of Genetics, Cell- and Immunobiology, Semmelweis University, Budapest, Hungary; 2 Department of Medical Biology, Medical School, Pecs University, Pecs, Hungary; 3 János Szentágothai Research Centre, University of Pecs, Hungary; 4 Endocrine Studies, Centre of Excellence, Pecs University, Pecs, Hungary; 5 Department of Obstetrics and Gynaecology, Medical School, Pecs University, Pecs, Hungary; 6 MTA - PTE Human Reproduction Research Group, Pecs, Hungary; 7 Institute of Bioanalysis, Medical School, Pecs University, Pecs, Hungary.

## Abstract

Multiple pregnancy is a risk for prematurity and preterm birth. The goal of assisted reproduction is to achieve a single pregnancy, by transferring a single embryo. This requires improved methods to identify the competent embryo. Here, we describe such a test, based on flow cytometric determination of the nucleic acid (PI+) containing extracellular vesicle (EV) count in day 5 embryo culture media. 88 women undergoing IVF were included in the study. More than 1 embryos were transferred to most patients. In 58 women, the transfer resulted in clinical pregnancy, whereas in 30 women in implantation failure. In 112 culture media of embryos from the “clinical pregnancy” group, the number of PI+ EVs was significantly lower than in those of 49 embryos, from the “implantation failure” group. In 14 women, transfer of a single embryo resulted in a singleton pregnancy, or, transfer of two embryos in twin pregnancy. The culture media of 19 out of the 20 “confirmed competent” embryos contained a lower level of PI+ EVs than the cut off level, suggesting that the competent embryo can indeed be identified by low PI+ EV counts. We developed a noninvasive, simple, inexpensive, quick test, which identifies the embryos that are most likely to implant.

The efficiency of embryo implantation is surprisingly low in humans. This can either be attributed to the high rate of chromosomally abnormal embryos, or to uterine factors, if the embryo is chromosomally normal. Ideally, a competent embryo should have good chances to implant into a receptive endometrium. However, successful outcome is also related to the age of the mother. The percentage of aneuploidy embryos increases with maternal age, reaching 80% over the age of 40^1^,^2^,^3^,^4^, and many of these chromosomally abnormal embryos fail to implant^5^,^6^,^7^,^8^,^9^, which explains that according to a rather conservative estimate only 50% of human conceptions will result in pregnancy^10^.

In an attempt to increase the chances of pregnancy in infertile women, many *in vitro* fertilization (IVF) centres transfer more than one embryos. This in turn also increases the hazard of twin pregnancies. Multiple pregnancies are among the most common causes of preterm birth, along with the increased risk for prematurity. Therefore, it would be of importance to select the embryo that is most likely to implant and to transfer that particular embryo only.

Enormous efforts have been devoted to finding the appropriate method to identify the competent embryo. Selection, based on the morphological features of the embryo^11^,^12^,^13^ is highly prone to subjectivity^14^. Morphokinetic measurements provide more objective data^15^,^16^. By using time-lapse imaging, the development of the embryo can be observed in a closed system, and thus, in contrast to morphological evaluation, in this case the culture conditions are stable. Some studies reported an elevated pregnancy rate when morphokinetic parameters were used for embryo selection, however, large randomized trials are still missing^17^.

Invasive methods, such as pre-implantation genetic screening for aneuploidy involve certain risks, since biopsy might negatively influence further development of the embryo^18^. Randomized trials performed with cleavage stage embryos did not show a beneficial effect on implantation and pregnancy rates^19^,^20^,^21^, partly because of chromosomal mosaicism, - the blastomere analysed, does not reflect the situation in the whole embryo^22^, or because since cleavage stage embryos are most vulnerable to invasive interventions, the biopsy itself might have contributed to the failure to show positive results^18^.

Analysis of embryo culture media is based on detection of changes in the spent embryo culture medium that would reflect the functional state and vitality of the embryo^23^,^24^,^25^,^26^. Recently, Monstko, *et al*.^27^ reported a 100% identification of non-viable embryos by the presence of a haptoglobin fragment in embryo culture medium. Although only 55% of successfully implanting embryos could be identified, this approach would still be a useful tool for embryo selection. However liquid chromatography together with mass spectrometry can hardly be expected to become available for routine use in IVF centres. Unfortunately, tests detecting the glucose or amino acid metabolism or oxygen consumption of the embryo also require sophisticated equipment. Therefore, they are not suitable for high throughput routine screening.

The ideal test should be non-invasive, simple and quick, so that it could be performed immediately, before fresh transfer.

Tannetta *et al*. suggested that preimplantation embryos might produce extracellular vesicles, however they point out that *in vitro* fertilization (IVF) culture media alone contains high levels of EVs, possibly from the nutrients supplemented for embryo growth^28^.

Extracellular vesicles (EVs) are phospholipid bilayer enclosed structures which are constitutively produced by both eukaryotic and prokaryotic cells. Among EVs exosomes are the smallest particles, with a diameter of approximately 100 nm or less. Microvesicles range from 100 to 800 nm and apoptotic bodies are larger than 1000 nm. On the basis of their specific exofacial and intra-vesicular molecular pattern (DNA, RNA and protein), EVs play important role in intercellular communication, both in physiological and pathological processes. EVs are detectable in body fluids including peripheral blood, urine, cerebrospinal fluid, synovial fluid, or amniotic fluid. Although most if not all cell types can produce any subpopulations of EVs, the different vesicles are induced by various stimuli^29^,^30^. Several methods are used for the assessment of the size distribution, morphology, molecular pattern, and cellular origin of EVs. The most often used detection techniques are Western blotting, nanoparticle tracking analysis (NTA), tunable resistive pulse sensing (TRPS), electron microscopy (EM) and flow cytometry.

The latter is an ideal, high-throughput method, suitable for multiparametric characterization of EVs^31^.

This study has set the goals i) to characterize embryo-derived EVs in embryo culture media by flow cytometry, ii) to correlate the characteristics of embryo-derived EVs with implantation potential and iii) to prove the applicability of the embryo-derived EVs as biomarkers in the prediction of the implantation potential.

## Methods

### Patients, sample collection

Eighty eight unselected infertile patients enrolled in the IVF program, were included in the study. The study was approved by the Human Reproduction Committee of the Hungarian Medical Research Council and the Ethical Committee of Pecs University. Informed consent was obtained from each patient. All methods were performed in accordance with the relevant guidelines and regulations.

For controlled ovarian hyperstimulation follicles were sycnhronized with the GnRh agonist triptorelin (Gonapeptyl; Ferring^®^, Germany) in either a long or short protocol. Follicle stimulation was performed with individual dosages of rFSH (Gonal-F; Serono^®^ Aubonne, Switzerland), varying from 112 to 225 IU per day. Final oocyte maturation was induced by injection of 250 μg of hCG (Ovitrelle; Serono^®^ Aubonne, Switzerland). Oocytes were harvested 36 hours later by ultrasonographically guided puncture of the follicles. Fertilization was performed with intracytoplasmatic sperm injection (ICSI) if sperm count was less than 15 M/ml, or the maternal age was higher than 35 years or if the number of the previous treatment cycles of the patient were more than two.

Embryos were cultured individually for 3 days under oil in G-1 medium. Then the medium was replaced by 40 μl of G-2 medium and the embryos were further cultured under oil till day 5. In the morning of day 5 (the day of the transfer) as much as possible of the spent medium was collected, and stored at −80 °C until used for EV determination. Oil (Ovoil), G-1 and G-2 medium were purchased from Vitrolife^®^, Göteborg, Sweden.

Embryos were transferred 3 or 5 days after the oocyte retrieval, however, only day 5 media from embryos transferred on day 5 were included in this study. The embryos to be transferred were selected by morphological criteria, using the Istambul Consensus embryo scoring system of ESHRE^32^. If possible, only expanded blastocysts with Grade 1 ICM and TE were transferred.

Implantation was confirmed by ultrasonography four weeks after the transfer. Clinical pregnancy is defined by the presence of foetal heartbeat, and implantation failure by the lack of the former, together with the lack of beta hCG on week 2 after the transfer.

### Flow cytometry

Measurements were carried out using a BD FACSCalibur (BD Biosciences) flow cytometer. All the FACS data were analyzed with CellQuestPro software. The instrument settings and gates were defined by Megamix-Plus SSC beads (Biocytex, France) and were optimized with 1 μm Silica Beads Fluo-Green Green (Kisker Biotech GmbH & Co; Steinfurt, Germany). (Supplementary Figure 1). The single-platform flow cytometric determination of the absolute number of EVs was performed by adding internal counting standard beads (Sysmex Partec GmbH; Germany) to IVF conditioned medium samples. The absolute number of EVs was calculated using the following formula:


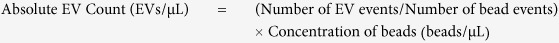


For Annexin V staining 2 μl IVF conditioned medium was diluted with 250 μl annexin binding buffer (BD Biosciences, San Jose, USA) and incubated for 10 minutes at room temperature with 1 μl phycoerythrin conjugated AnnexinV (BD Biosciences, San Jose, USA). Fifty μl Count Check Beads (Sysmex Partec GmbH) was also added for determination of the number of EVs. To confirm the presence of EVs, we applied Triton-X differential detergent lysis using a final concentration of 0.1%. Only events that disappeared in the presence of 0.1% Triton-X 100 were considered as vesicles^33^,^34^.

Propidium iodide (PI) was used for the labelling of the nucleic acid content of embryo-derived EVs. PI is a fluorescent intercalating agent which is commonly used as a DNA stain for flow cytometry. Twenty five μl of conditioned embryo culture media was used for the measurements. Embryo culture media were incubated for 15 minutes at room temperature with 100 μl 4% formaldehyde (from paraformaldehyde (PFA) solution. At the end of the incubation, 150 μl filtered PBS and 1 μl PI solution (50 μg/ml) and 50 μl Count Check beads (Sysmex Partec GmbH) were added to the sample. FACS analysis was carried out within 30 minutes after PI staining. Unstained samples were used for the detection of autofluorescence. Empty G-1 and G-2 medium and oil for embryo culture were also incubated together PI dye for determination of the specificity of DNA labelling method. (Supplementary Figure 2). The single-platform flow cytometric determination of the absolute number of EVs was performed by adding internal counting standard beads (Sysmex Partec GmbH; Germany) to IVF conditioned medium samples.

### Confirmation of the presence of EVs in IVF medium by using transmission electron microscopy

Pooled samples (2 IVF medium) were used for transmission electron microscopic analysis. After centrifugation of pooled samples (20500 g, 20 minutes), the supernatant was carefully removed and the pellet was fixed at room temperature for 30 min with 4% formaldehyde, in 0.01 M phoshate buffer (PBS) at pH 7.4). After washing with phosphate buffer several times, the preparations were postfixed in 1% OsO_4_ (Taab; Aldermaston, Berks, UK) for 30 min. Following washing with distilled water, the pellets were dehydrated in graded ethanol, including block-staining with 2% uranyl acetate in 70% ethanol for 30 min, and embedded in Taab 812 (Taab). Ultrathin sections were examined in a Hitachi 7100 transmission electron microscope (Hitachi Corporation, Japan). Electron micrographs were taken at the same magnification (40,000).

### Statistical analysis

The distribution of the PI positive (PI+) EVs was tested with the Kolgomorov-Smirnov’s test, which showed a nonparametric distribution. To determine differences between the groups the Mann-Whitney test was used. Receiver operating curve (ROC) analyses was used evaluate the diagnostic ability of test, and the optimal cut-off score was set by the Youden index^35^. P-values < 0.05 were considered as statistically significant.

## Results

### Cultured human embryos release EVs

**Figure 1:**
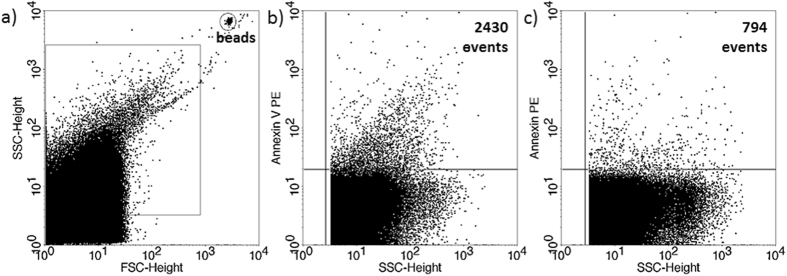
Annexin V staining of embryo-derived EVs in embryo culture medium. (**a**) Representative FSC-SSC dot plot shows the size distribution of EVs in embryo culture medium. (**b**) Representative dot plot shows the phycoerythrin (PE) fluorescence of EVs after AnnexinV-PE labelling. (**c**) Representative dot plot shows the PE fluoresce after Triton-X 100 differential detergent lysis.

**Figure 2:**
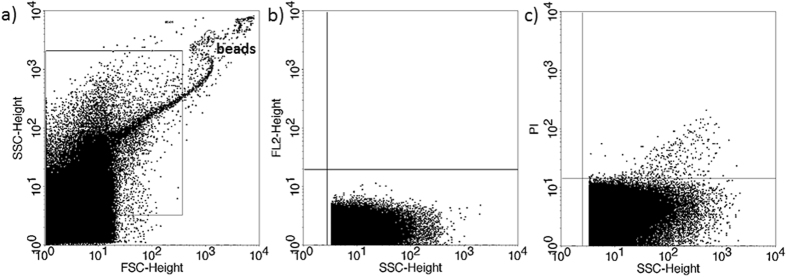
Propidium iodide (PI) staining of embryo-derived EVs in embryo culture medium. (**a**) Representative FSC-SSC dot plot shows the size distribution of EVs in embryo culture medium. (**b**) Representative dot plot shows the autofluorescence of embryo culture medium in FL2 channel (embryo culture medium + 4% formaldehyde solution +PI, without EVs. (**c**) Representative dot plot shows the PI fluorescence of 4% formaldehyde fixed embryo-derived EVs in embryo culture medium.

The presence of EVs in culture media of *in vitro* fertilized human embryos was demonstrated by flow cytometry. Although phosphatidylserine is predominantly located in the inner leaflet of the plasma membrane, it is externalized in EVs, thus it could be stained by Annexin V. To confirm the flow cytometric detection of Annexin V positive EVs, we used Triton-X differential detergent lysis. Only the events sensitive to 0.1% Triton-X 100 were considered as EVs (Fig. 1). Nucleic acid-containing embryo-derived EVs could be detected by Propidium iodide (PI) after fixation of vesicle membrane (Fig. 2). The presence of EVs was also confirmed with transmission electron microscopy (Supplementary Figure 3).

### The number of PI+ EVs is significantly lower in culture media of competent-, than in those of failed embryos

Eighty eight infertile women were included in the study. To increase the chances of viable pregnancy, according to the national legislation in most cases two or three embryos were transferred simultaneously.

Two groups were formed according to the outcome;

1. Implantation failure N = 30.
2. clinical pregnancy N = 58.

**Figure 3:**
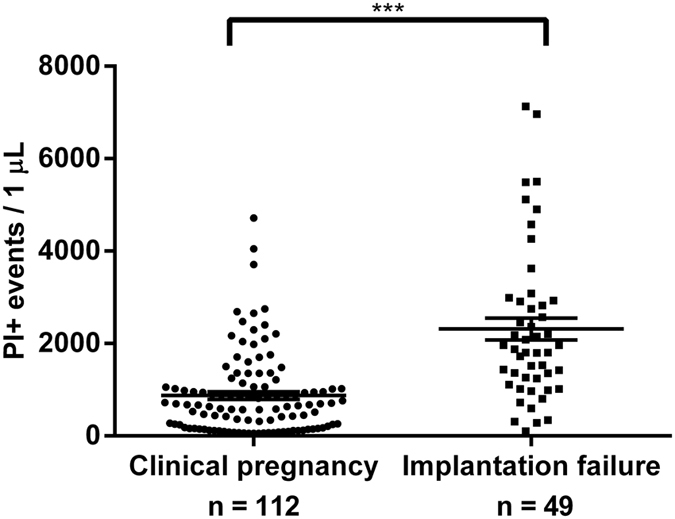
Propidium iodide (PI)+ EVs in culture medium of embryos resulting in clinical pregnancy or implantation failure. ***P < 0.001.

The number of PI+ EVs was significantly (p < 0.001) lower in the 5^th^ day culture media of embryos from the “clinical pregnancy” group (N = 112), than in those of embryos, that failed to implant (N = 49) (Fig. 3).

### Identification of competent embryos by determining the number of PI+ EVs in day 5 culture medium

Transfer of 112 embryos to 58 women resulted in 64 implantations. Two embryos were transferred to 45 women, three to 5 women and a single embryo to 8 women. (One sample was used for other purposes, not included in the statistics).

**Figure 4:**
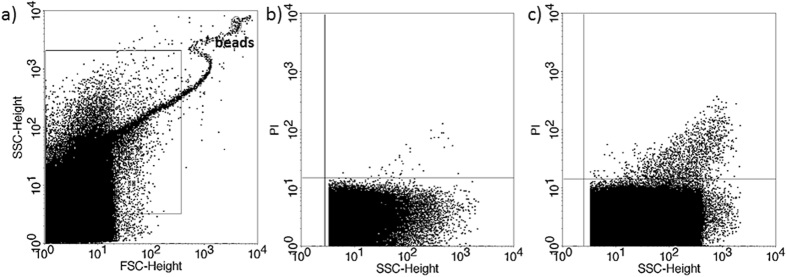
Propidium iodide (PI) staining of embryo-derived EVs in embryo culture medium. Two transferred embryos resulting in singleton pregnancy. (**a**) Representative FSC-SSC dot plot shows the size distribution of EVs in embryo culture medium. (**b**,**c**) dot plots show the PI fluorescence of EVs transferred to the same mother.

**Figure 5:**
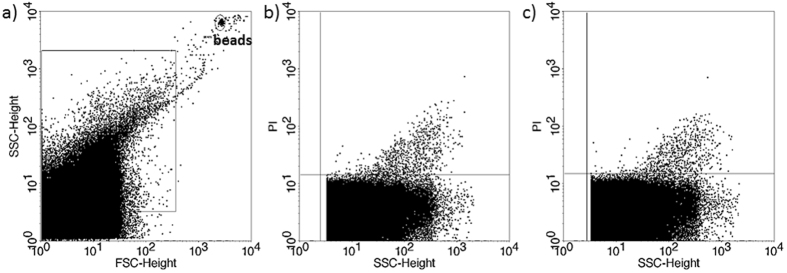
Propidium iodide (PI) staining of embryo-derived EVs in embryo culture medium. Two transferred embryos resulting in pregnancy failure. (**a**) Representative FSC-SSC dot plot shows the size distribution of EVs in embryo culture medium. (**a**,**c**) dot plots show the PI fluorescence of EVs transferred to the same mother.

If transfer of two embryos resulted in a singleton pregnancy, the culture media from one of the transferred embryos contained considerably less PI+ EVs than the other (Fig. 4), while, if transfer of two embryos resulted in implantation failure, culture media of both embryos contained a high number of PI+ EVs (Fig. 5).

**Figure 6:**
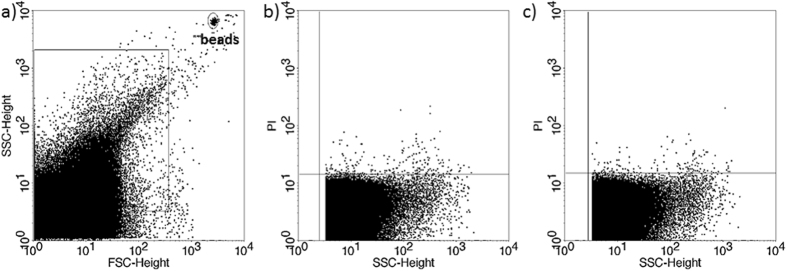
Propidium iodide (PI) staining of embryo-derived EVs in embryo culture medium. Two transferred embryos, resulting in twin pregnancy. (**a**) Representative FSC-SSC dot plot shows the size distribution of EVs in embryo culture medium. (**b**,**c**) dot plots show the PI fluorescence of EVs transferred to the same mother.

In 6 cases transfer of two embryos resulted in twin pregnancy. The culture media of both embryos contained a low number of PI+ EVs (Fig. 6).

Based on these data we assume that the embryos releasing lower number of PI+ EVs were the ones that implanted.

When transfer of more embryos resulted in singleton pregnancies, PI+ EV counts were lower in one of the embryo culture media than in the others.

**Figure 7:**
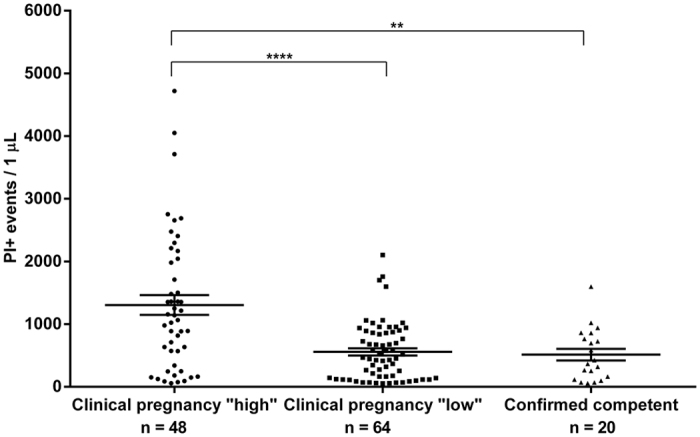
Culture media of embryos from clinical pregnancies with higher (N = 48) or lower (N = 64) number of PI+ EVs. ***p < 0.001 ****p < 0.0001.

When culture media with lower number of PI+ EVs were analysed against culture media of embryos with higher number of PI+ EVs within the “clinical pregnancy” group, there was a significant difference (p < 0.001) between the lower PI+ EV and higher PI+ EV groups (Fig. 7). In 8 cases, transfer of a single embryo resulted in a singleton pregnancy, and in 6 cases, the transfer of two embryos ended up in a twin pregnancy. All of these “confirmed competent” embryos contained low PI+ EV counts (Fig. 7). This confirms, that the competent embryo can indeed be identified by lower PI+ EV counts.

These data also suggest, that if there is a marked difference in the PI+ EV counts in the culture media of different embryos from the same mother, it is the embryo with lower number of nucleic acid-containing EVs that is more likely to implant.

### A cut off level for identifying the competent embryos

#### ROC analysis was performed using different data sets

**Figure 8:**
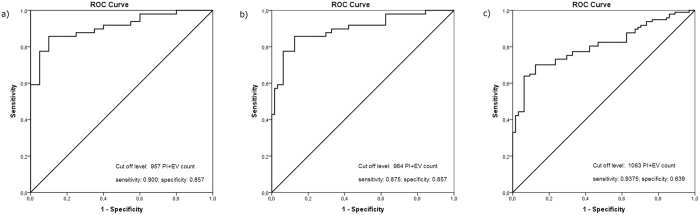
Evaluation of optimal cut-off score and the diagnostic ability of test by ROC analysis. (**a**) Data of confirmed competent embryos are plotted in function of data from implantation failure. (**b**) Data from presumed competent embryos (giving the lowest PI+ EV values among embryos from the same mother) are plotted against data from implantation failure. (**c**) Data from presumed competent embryos are plotted against data from presumed incompetent embryos in the clinical pregnancy group, plus data from the implantation failure group.

Plotting data of confirmed competent embryos versus data from implantation failure (Fig. 8a) yielded a cut off level of 957 PI+ EV count that corresponded to a maximum specificity and sensitivity. The AUC (area under the curve) was 0.91, (95% CI: 0.842–0.978). Sensitivity; 0.9 specificity; 0.857.

Plotting data from presumed competent embryos (giving the lowest PI+ EV values among embryos from the same mother) against data from implantation failure (Fig. 8b) resulted in a cut off level of 964. The AUC was 0.899 (95% CI: 0.837–0.960) Sensitivity; 0.875 specificity; 0.857.

Plotting data from presumed competent embryos against data from presumed incompetent embryos in the clinical pregnancy group, plus data from the implantation failure group (Fig. 8c). The cut off value was 1063, the AUC was 0.806 (95% CI: 0.740–0.873) Sensitivity; 0.9375 specificity; 0.639.

The AUC is a measure of how well a parameter can distinguish between two diagnostic groups. The present values show a very good distinction.

When the cut off was set at 964 PI+ EVs, the culture media of 8 (12.5%) out of 64 potentially competent embryos contained a higher number of PI+ EVs than the cut off, whereas in 7 (14%) of the 49 samples from the implantation failure group PI+ EV counts fell below the cut off level. The culture media of 19 out of the 20 “confirmed competent” embryos contained lower PI+ EV counts than the cut off level.

Based on these data, the embryo to be transferred should be the one with the lowest PI+ EV count among all the embryos from the same mother. The question is, how many of such embryos have a higher PI+ EV count, than the cut off level, in other words; what is the chance for false positivity. In the present study, 8 (12.5%) of the 64 supposedly competent embryos were falsely diagnosed. Therefore, when an IVF specialist transfers the embryo with the lowest PI+ EV count, the chances of implantation are close to 90%, provided the maternal side is receptive.

## Discussion

Due to the increasing rate of infertility in developed countries, assisted reproduction has become a routine procedure. Transferring a single competent embryo, is crucial for avoiding multiple pregnancies, however to achieve this goal, no reliable method is available to identify the competent embryo at present. A major disadvantage of aneuploidy screening is its invasive nature^18^, selection based on embryo morphology^11^,^12^,^13^ is subjective and morphology does not always correlate with implantation potential^14^. Analysis of embryo culture media based on the supposedly different metabolic activity of competent and incompetent embryos is time consuming and requires sophisticated equipment^23^,^24^,^25^,^26^,^27^.

Here, we describe a non-invasive, simple, inexpensive, and most importantly quick test, to identify the embryos that are most likely to implant in a receptive endometrium. Less than 1 h is required to test 15 embryo culture media, thus the test can easily performed before fresh transfer. A clear disadvantage of the method is, that a flow cytometer is an expensive equipment. On the other hand; given the short time required for performing the test, the IVF centre does not necessarily be equipped with a flow cytometer. Measurements can be done in a nearby flow cytometric core facility. We used culture media of day 5 embryos, because if the embryo is cultured *in vitro* till it develops into blastocyst, the developmental uncertainties of cleavage stage embryo development can be eliminated. Furthermore, the implantation potential of the blastocyst seems to be better than, that of cleavage stage embryos. A single-blastocyst transfer is much more likely to result in a singleton live birth, than transfer of a single good-quality cleavage-stage embryo on day 3^12^,^36^. Therefore, simply allowing the embryo to reach the blastocyst stage, might improve the implantation rate in a fresh transfer.

In this study we showed that *in vitro* fertilized embryos release detectable numbers of extracellular vesicles, into the culture media, and a part of these contain nucleic acid detectable by PI staining. The origin of the nucleic acid has not been investigated in this study, but a possible explanation could be that it is released due to cell damage, thus the higher number of nucleic acid containing EVs reflect the impairment of the embryo.

We demonstrated that when the transfer of multiple embryos resulted in singleton pregnancies, PI+ EV counts were lower in one of the embryo culture media than in the others. Based on these data we assumed that the embryos releasing lower number of PI+ EVs were the ones that implanted. To prove this concept, we collected the culture media of embryos, with a known outcome of the transfers. Culture media from twenty embryos from either single transfers resulting in clinical pregnancies, or from transfer of two embryos resulting in twin pregnancies were tested. Nineteen of these 20 “confirmed competent” embryos produced lower than the cut-off PI+ EV numbers in the culture media, suggesting that determining the number of PI+ EVs in the culture medium is indeed suitable for identifying the competent embryo.

Considering the relatively small sample size, the sensitivity and specificity of the test are acceptable.

Of note; while in the cases resulting in clinical pregnancies, both the maternal side and - at least one - of the embryos must have been competent; in women, with failed implantation, the reason for the failure could be either embryonic, or maternal or both. Therefore, when we compare the PI+ EV content of presumably competent embryos to that of embryos from the implantation failure group, on the latter side we are dealing with a heterogeneous population. In addition to the incompetent embryos, competent embryos might also have been present, which failed to implant, because of endometrial receptivity problems.

The other problem concerns the methodology. Although the method is very simple, the inherent problems of flow cytometry –if not properly handled might cause inconsistent results. Even though flow cytometry is a common method for characterizing EVs, it has many difficulties. Different instruments use different counting methods. There are two trends in flow cytometric absolute counting: 1) the volumetric- and 2) the microbead-based absolute counting. In our experiments microbead-based assessments were applied for FACSCalibur cytometer. In these cases the appropriate sample handling is very important to avoid false results or artefacts. Our data indicate that during microbead-based absolute counting procedures the same type of calibration beads must be used in order to avoid inter-assay variations. In order to achieve the highest sensitivity we also suggest the detection of background noise by the analysis of PI containing empty culture media (Supplementary Figures 1 and 2).

If these problems are taken in account and properly dealt with, this system can be useful in identifying the competent embryo right before fresh transfer.

## Additional Information

**How to cite this article:** Pallinger, E. *et al*. A simple and rapid flow cytometry-based assay to identify a competent embryo prior to embryo transfer. *Sci. Rep.* **7**, 39927; doi: 10.1038/srep39927 (2017).

**Publisher's note:** Springer Nature remains neutral with regard to jurisdictional claims in published maps and institutional affiliations.

## Supplementary Material

Supplementary Information
