# Supplementary material for: A simple and rapid flow cytometry-based assay to identify a competent embryo prior to embryo transfer
Source: Sci Rep. 2017 Jan 6;7:39927. doi: 10.1038/srep39927 (PMC5216337; doi:10.1038/srep39927)
Supplement: Supplementary Information [file srep39927-s1.pdf]

A simple and rapid flow cytometry-based assay to identify a competent embryo prior to embryo transfer.

Eva Pallinger[1], Zoltan Bognar[2,5,7], Jozsef Bodis [3,4,7], Timea Csabai [2,5,7], Nelli Farkas [6]

Krisztina Godony[3] , Akos Varnagy[3,4,7], Edit Buzas[1], Julia Szekeres-Bartho* [2,4,5,7]

1 Department of Genetics, Cell- and Immunobiology, Semmelweis University, Budapest

2 Department of Medical Biology, Medical School, Pecs University,

3 Department of Obstetrics and Gynaecology, Medical School, Pecs University

4 MTA - PTE Human Reproduction Research Group,

5 János Szentágothai Research Centre, University of Pecs, Hungary.

6 Institute of Bioanalysis, Medical School, Pecs University

7 Endocrine Studies, Centre of Excellence

Correspondence to;

Julia Szekeres-Bartho szekeres.julia@pte.hu

Department of medical Biology

Medical School Pecs University,

12 Szigeti Street, 7624 Pecs, Hungary

Supplementary Fig. 1

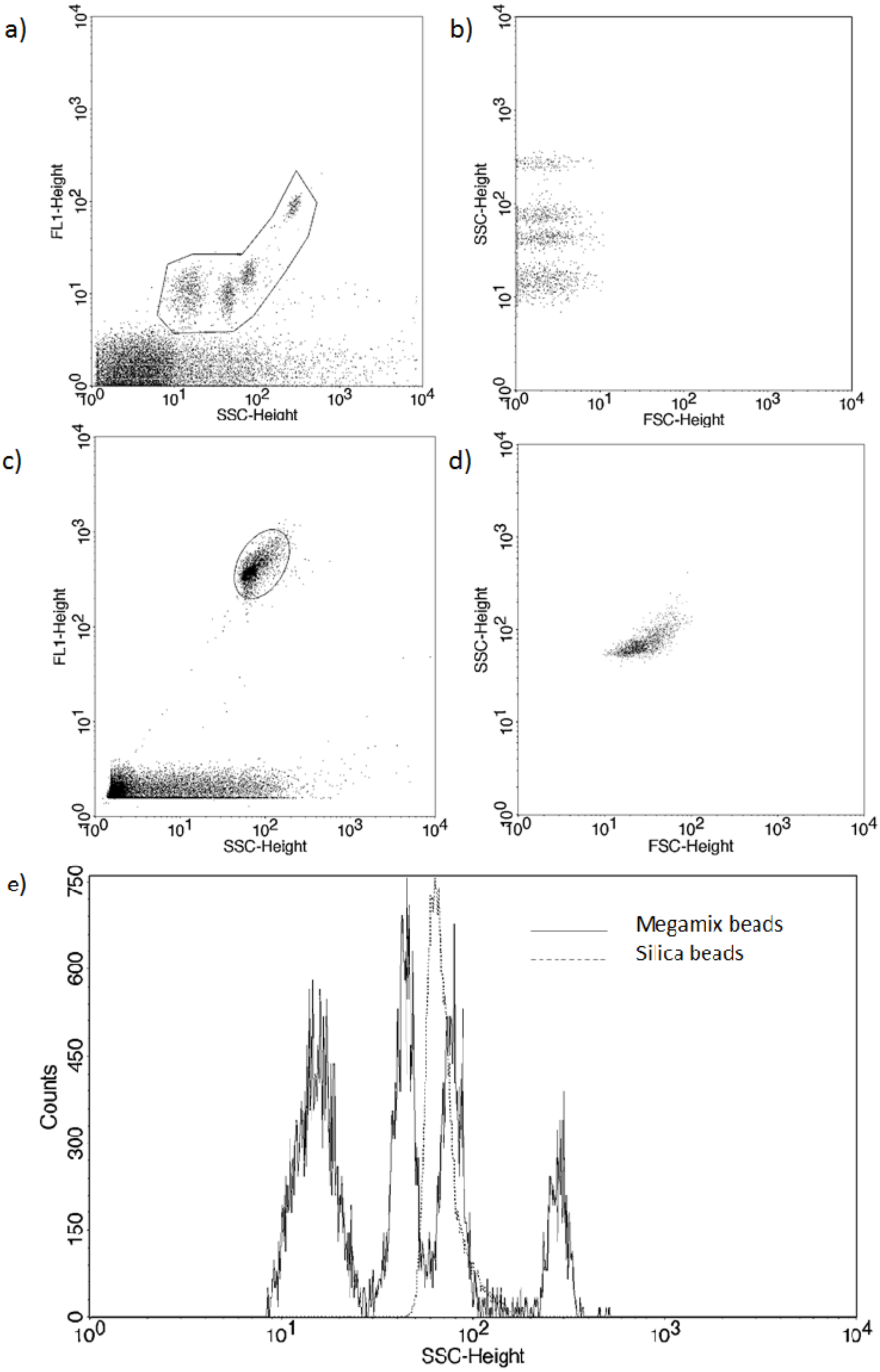

Supplementary Fig.2

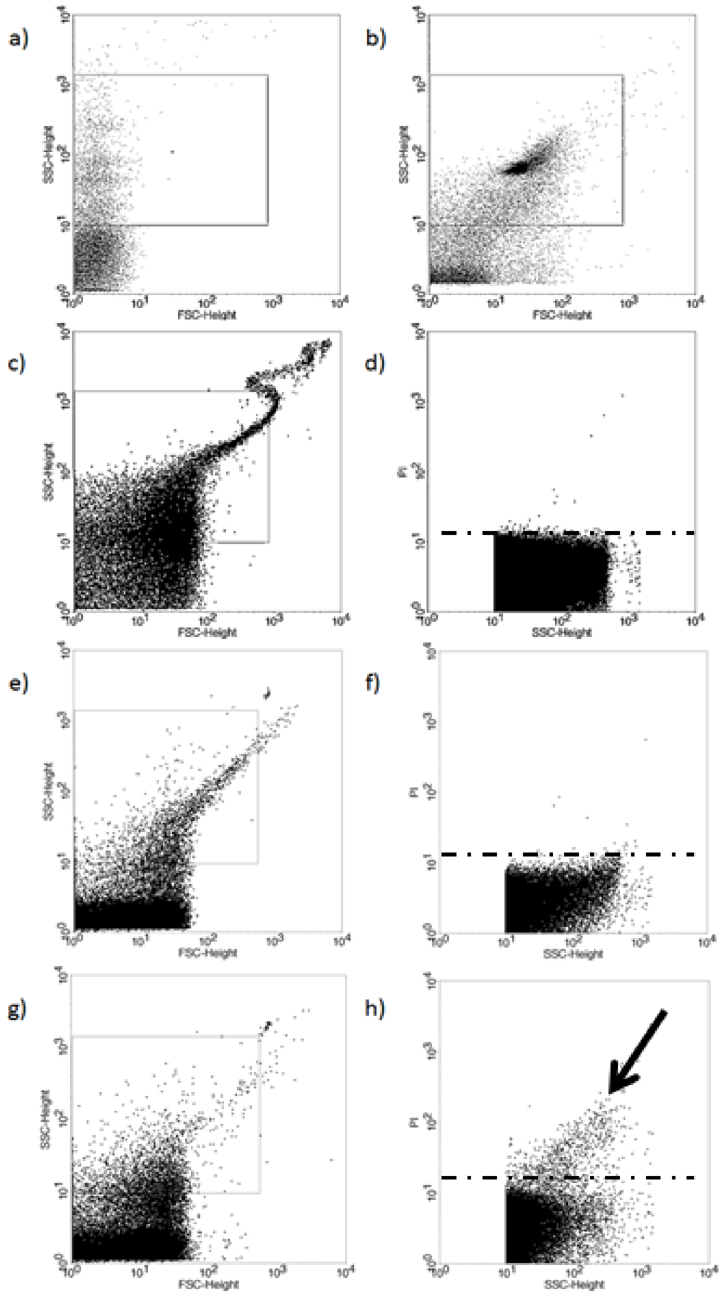

Supplementary Fig. 3

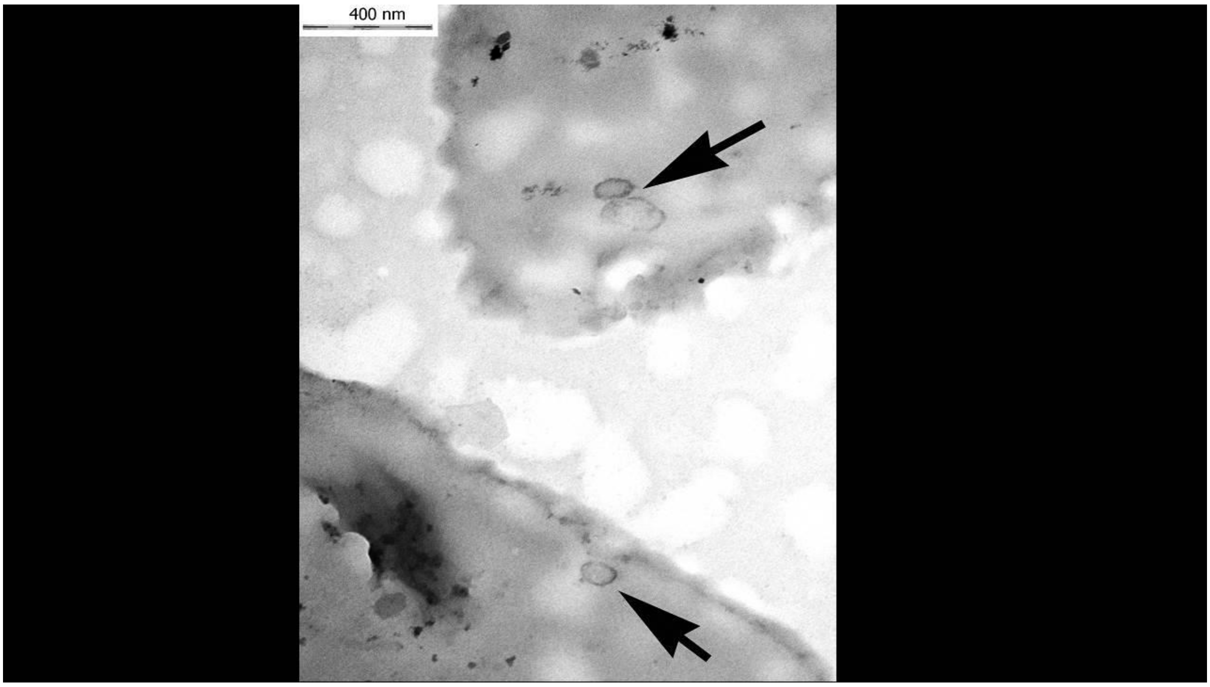

Legends to supplementary figures

Supplementary figure 1: Flow cytometer setup

Fluorescent Megamix-Plus SSC polystyrene beads (Biocytex, France) and Silica Beads Fluo-Green (Kisker Biotech GmbH & Co; Steinfurt, Germany) were used for optimization of cytometer settings. Although scatter parameters are determined by the morphology, size, absorption and refractive index of particles, these are also depending on laser characteristics. In the case of extracellular vesicles both scatter parameters (FSC, SSC) are primarily related to vesicle diameter. FACSCalibur (BD biosciences, USA) cytometers use photomultiplier tubes for the detection of SSC signals and photodiodes for FSC detection. It means that SSC parameter is more sensitive in FACSCalibur, so it can be used for extracellular vesicles.

Comparative dot plots of SSC on X-axis vs. FL1 fluorescence on Y-axis show the fluorescence intensities of megamix (a) or silica (c) calibration beads. Dot plots of FSC on X-axis vs. SSC on Y-axis show the size distribution of megamix (b) or silica (d) calibration beads. Representative overlay histogram (e) compares the SSC parameters of megamix and silica beads. Peaks of solid curve represent the relative size of megamix bead populations (160 nm, 200 nm, 240 nm, 500 nm).

Supplementary figure 2: Gating strategy and confirmation of PI staining

EV population was defined by gating on FSC-SSC dot plot. Gating strategy based on FSC-SSC dot plots of Megamix-Plus SSC polystyrene beads (Biocytex, France) (a) and Silica Beads Fluo-Green (Kisker Biotech GmbH & Co; Steinfurt, Germany) (b).

DNA staining was performed after paraformaldehyde fixation, so we could not apply differential detergent lysis for verifying the vesicles. In order to prove the staining specificity we calculated the fluorescence background noise. Propidium iodide (PI) stained embryo culture oil and unconditioned embryo culture medium was analysed by flow cytometry using the same instrument settings then in the case of IVF sample measurements. Dot plots of FSC on X-axis vs. SSC on Y-axis show the particle size distribution of embryo culture oil (c) or unconditioned embryo culture medium (e). Comparative dot plots of SSC on X-axis vs. FL2 fluorescence on Y-axis show the PI fluorescence background of embryo culture oil (d) or unconditioned embryo culture medium (f). g) and h) dot plots represent the size distribution (FSC vs. SSC) and the

PI fluorescence (SSC vs. FL2) of conditioned embryo culture medium. Black arrow on h) dot plot indicates the PI+ MVs.

Supplementary figure 3. Transmission electron microscope image of embryo-derived EVs in embryo culture medium

The arrows indicate extracellular vesicles of 100 to 200 nm in culture medium of embryos. Four embryo culture media (40 μl each) were pooled, centrifuged at 12500g for 20 min. The pellets were embedded for electron microscopy.
